# Moving forward: why responding to migration, mobility and HIV in South(ern) Africa is a public health priority

**DOI:** 10.1002/jia2.25137

**Published:** 2018-07-19

**Authors:** Jo Vearey

**Affiliations:** ^1^ African Centre for Migration & Society University of the Witwatersrand Wits South Africa; ^2^ Centre of African Studies University of Edinburgh UK

**Keywords:** migration, mobility, HIV, southern Africa, Global Compact, SADC

## Abstract

**Introduction:**

Global migration policy discussions are increasingly driven by moral panics – public anxiety about issues thought to threaten the moral standards of society. This includes the development of two Global Compacts – agreed principles to guide an international response – for (1) “Refugees” and (2) “Safe, Regular and Orderly Migration.” While the need to address migration and health is increasingly recognized at the global level, concerns are raised about if this will be reflected in the final Compacts. The Compacts focus on securitization, an approach that aims to restrict the movement of people, presenting potentially negative health consequences for people who move. Globally, concern is raised that migration‐aware public health programming initiatives could be co‐opted through a global health security agenda to further restrict movement across borders. This is particularly worrying in the Southern African Development Community (SADC) – a regional economic community associated with high levels of migration and the largest population of people living with HIV globally; this case is used to explore concerns about the health implications of the Global Compacts.

**Discussion:**

Current HIV responses in SADC do not adequately engage with the movement of healthcare users within and between countries. This negatively affects existing HIV interventions and has implications for the development of universal HIV testing and treatment (UTT) programmes. Drawing on literature and policy review, and ongoing participant observation in policy processes, I outline how Global Compact processes may undermine HIV prevention efforts in SADC.

**Conclusions:**

The global health imperative of developing migration‐aware and mobility‐competent health responses must not be undermined by moral panics; the resultant international policy processes run the risk of jeopardizing effective action at the local level. Globally, migration is increasingly recognized as a central public health concern, providing strategic opportunities to strengthen public health responses for all. Without mainstreaming migration, however, health responses will struggle. This is particularly concerning in SADC where HIV programmes – including UTT initiatives – will struggle, and key health targets will not be met. Globally, contextually appropriate migration‐aware responses to health are needed, including and a specific focus on HIV programming in SADC.

## 1 Introduction

While health has long been considered an essential component of human and economic development, the health of migrants has remained in the shadows of key global health, migration, and development dialogues and processes, and many migrants still lack access to affordable health services. (IOM, 2017: p. 4)

In spite of recent calls at the global level to improve responses to migration and health, and the development of a global research agenda to support this 1, 2, 3, 4, the “unfinished agenda of migrant health for the benefit of all” 5, 6 remains a glaring gap in current global, regional and national policy discussions. To further complicate this situation, attempts to develop interventions on migration and health – at all levels – may be undermined by the global migration policy terrain. Following the 2016 “New York Declaration on Refugees and Migrants” 7, two Global Compacts are due to be finalized and released in the second half of 2018 – the “Global Compact on Refugees,” and the “Global Compact on Safe, Regular and Orderly Migration.” Global Compacts refer “to an agreement between states on matters of common interest of concern,” and provide opportunities for determining how member states will “conduct themselves in the future” – in this case in relation to the management of migration 8. Two discrete motivations for engaging with migration at a global level currently exist – one from a right to health, wellbeing, and public health perspective 1, 3, and the other from a securitization and restriction of movement approach 8, 9. The resulting tensions within the global community present multiple challenges for the development of improved responses to migration and health.

In this commentary, I explore the implications of the current global migration policy terrain for the Southern African Development Community (SADC), a regional economic community made up of 15 member states that is associated with high levels of both internal (within country) and cross‐border (between country) migration and a high communicable disease burden, including the largest population of people living with HIV globally 10, 11, 12, 13. With the aim of offering suggestions for ways to mobilize a regional response to migration and HIV in SADC, I outline the challenges – and strategic opportunities – that result from the current global migration policy terrain. This is done by drawing on a review of research and policy associated with migration and HIV in SADC (13 and Table 1) and my ongoing participant observation within various global, regional and national policy processes.

**Table 1:** An overview of key global and regional migration and health policy processes (expanded on from 3, 12)

Year	Process
2003	WHO publishes International Migration, Health and Human Rights 54
	International Organization for Migration (IOM) Position Paper on Psychosocial and Mental Well‐Being of Migrants 55
2004	Migrant health for the benefit of all MC/INF/275 56
2006	African Union Executive Council. 2006. African Common Position on Migration and Development 32
	African Union Executive Council. 2006. The Migration Policy Framework for Africa 57
2009	Draft 2009 declaration on population mobility and communicable diseases and associated financing model (Southern African Development Community (SADC)) 40 (Oxford Policy Management, unpublished work)
2008	WHA Resolution 61.17 on the Health of Migrants 58
2010	2010 1st Global Consultation: The Health of Migrants – the Way Forward Madrid, Spain, 3 to 5 March 2010 28
	SADC HIV Cross Border Initiative 41
2012	TB in the Mines (TIMS) 59 and SADC TB in the mines 42
2015	IOM 106th Council Session: 26th Nov 2015, Geneva, Switzerland Advancing The Unfinished Agenda Of Migrant Health For The Benefit Of All – C/106/INF/15 5 High‐level Panel Discussion on Migration, human mobility and global health: a matter for diplomacy and intersectional partnership 6
2016	UN GENERAL ASSEMBLY: 9th May 2016 – High‐level Meeting on Addressing Large Movements of Refugees and Migrants Report of the Secretary‐General: In Safety and Dignity: Addressing Large Movements of Refugees and Migrants 60
	69th World Health Assembly: 27th May 2016 Technical Briefing on Migration and Health 61 Promoting the Health of Migrants. Report from the Secretariat 62
	UN General Assembly High‐level Meeting to Address Large Movements of Refugees and Migrants: 22nd Sept 2016 Side Event Report – Health in the Context of Migration and Forced Displacement 63
	3rd October 2016: New York Declaration for Refugees and Migrants Resolution adopted by the General Assembly on 19 September 2016 7
	Leaving no one behind: the imperative of inclusive development Report on the World Social Situation 2016 64
2017	January 2017 – 140th Session of the WHO Executive Board of the World Health Noted the WHO Secretariat report on Promoting the health of migrants 62 Adopted Decision EB140 9 – Promoting the health of refugees and migrants 65
	February 2017: 2nd Global Consultation – Health of Migrants: Resetting the Agenda 3
	17th May 2017: WHO Input to the 70th World Health Assembly – Draft framework of priorities and guiding principles. A70/24 66
	70th World Health Assembly – 30th May 2017 Adoption of WHA Resolution 70.15 Promoting the Health of Refugees and Migrants 67
	Global Compact Process IOM Thematic Paper: The Health Of Migrants: A Core Cross‐Cutting Theme 68
	IOM Migration Health Division – Thematic Paper Series MIGRATION HEALTH IN THE SUSTAINABLE DEVELOPMENT GOALS: “Leave No One Behind” in an increasingly mobile society 25
2018	142nd WHO Executive Board Meeting 71st World Health Assembly 109th IOM Council Global Compact on Refugees Global Compact on Safe, Regular and Orderly Migration
2019	144th WHO Executive Board session 72nd World health Assembly WHO Draft Global Action Plan on the Health of Refugees and Migrants to be submitted for consideration
2030	UN 2030 Agenda for Sustainable Development 26

## 2 Discussion

### 2.1 A global policy crisis?

As evidenced by the current Global Compact processes, we find ourselves in a world increasingly concerned with securitizing national borders and restricting the movement of people between nation states. Much of this focus on security is driven by moral panics – public anxiety about issues thought to threaten the moral standards of society – associated with migration, including human trafficking and the independent movement of women 9, and the so‐called “Migration Crisis” in Europe 14, 15, 16. Internationally, discussions on migration tend to ignore long‐established population movements within Global South contexts where forced migration and movement in search of improved livelihood opportunities are commonplace and outnumber similar movements in the Global North; the Southern African region is no exception 17. The current global discourses surrounding population mobility – that are fuelling morally panicked policy discussions – have negative impacts both for those who move, and for the development of improved responses to migration and health. Centrally, this includes the implementation of increasingly restrictive immigration policies, including further securitization of the borders of nation sates. In relation to health and wellbeing, historical perceptions of the migrant as the “diseased body”; as a carrier and transmitter of infectious diseases, particularly HIV; and, consequently, as a burden on the welfare state of receiving countries, are re‐emerging 18, 19, 20, 21. We need to remain vigilant and ensure that the re‐emergence of this discourse is not used to support securitization agendas as health status may (once again) be used to mediate the ability to legally cross national borders. Particularly worrying is that this may include an unwelcome return to a focus on the HIV status of people crossing borders.

The “draft zeros” of the two Global Compacts were released in early 2018 22, 23 and, while they do acknowledge health, concerns remain that health – and other social justice issues – will be left off the final agenda 9, 24. As a result, the final Global Compacts run the risk of calling for actions that ignore – or may even be in contravention of – existing approaches aimed at improving health for all, including the Sustainable Development Goals (SDGs) 25, 26 and the Global Compact for Universal Health Care 27. Table 1 summarizes the key international policy processes that have been engaging with migration and health, and the more recent processes associated with the Global Compacts. From a review of these processes, participation in both the 1st and 2nd Global Consultations on Migration and Health in 2010 and 2017 3, 28, and more recent engagement in global migration and health research initiatives, it becomes apparent that the progressive agenda being developed around migration and health stands the risk of being undermined by the Global Compacts process. With a clear focus on securitization and the restriction of movement, health‐related issues are being side‐lined in current global discussions. In addition, and particularly important for SADC, concerns have been raised that the loudest voices – those associated with the anti‐immigrant agendas of northern Europe, are driving the Global Compact processes, resulting in a global agenda calling for further restrictions on international migration which will be detrimental to other regions of the world, including the African continent 29, 30, 31. To this end, the African Union developed a draft Common African Position, that involved regional dialogues – including within SADC – in an attempt to ensure that the contextual realities of the continent are engaged with in the finalization of the Global Compacts 32. This Position paper – which makes reference to the importance of ensuring access to healthcare for migrants – was presented in draft form at the international preparatory meeting on the Global Compacts held in Mexico in December 2017, and has since been finalized and approved 33. How effective these interventions are, and whether health‐related concerns are emphasized in the final Compacts, remains to be seen.

### 2.2 Migration, mobility and HIV

In spite of the SADC region being associated with a long history of diverse population movements, current public health responses to HIV still fail to adequately engage with migration and the movement of healthcare users – both within and between countries 10, 11, 12. The result is a range of negative outcomes with serious implications for population health and HIV prevention, including challenges in initiating treatment, ensuring treatment continuity, and the associated risks for defaulting and drug resistance 34. In addition, the absence of evidence‐informed and migration‐aware responses to HIV has led to the continued scapegoating of migrants – particularly non‐nationals – as the carriers of HIV; the “diseased” migrant body is a long‐standing trope, and one that conjures up the idea of the migrant as a disease vector, whose movements are solely responsible for the spread of new HIV infections 18. Such imaginings conveniently absolve the state from its own shortcomings in terms of inadequate healthcare, health promotion and HIV prevention strategies. Rather, the state and their healthcare institutions blame internal and cross‐border movements for placing an excessive burden on the state.

Globally – and in SADC – key population groups currently targeted for HIV prevention initiatives are often highly mobile, including sex workers and men who have sex with men 13. Recent UNAIDS Gap Reports make the case for developing migration‐aware responses to HIV, acknowledging the importance of developing cross‐border initiatives and mainstreaming migration into national HIV strategic plans 13, 35, 36, 37. This has implications for the development and implementation of effective HIV prevention programming, including universal HIV testing and treatment (UTT) and pre‐exposure prophylaxis (PreP). A key challenge relates to how to do this: migration is a politically sensitive issue – associated with anti‐foreigner and xenophobic rhetoric, in spite of internal mobility being far more prevalent‐ and HIV (and health more broadly) is also a politically sensitive concern. Bringing together the interconnected concerns and agendas relating to migration and HIV is challenging and innovative approaches to this are required; migration is a central public health concern that – if approached carefully – can provide a strategic opportunity to strengthen responses to HIV for the benefit of all. For example, framing the development of responses to migration and HIV as a key entry point to improving health for all, for addressing health inequities, and for working towards universal health coverage, could assist in obtaining the political support needed to fund and support migration‐aware responses to HIV in SADC. This will have further positive impacts, namely in supporting activities for achieving global health targets, including the SDGs 26 and the UNAIDS 90‐90‐90 targets 38.

However, a key challenge exists – evidence suggests that SADC remains poorly equipped to initiate and manage the political discussions within and between member states that are required to develop appropriate regional responses to migration, mobility, and HIV 11, 39. This is partly due to the historical preference for the development of individual bilateral agreements between member states rather than regional responses 39. However, as presented in Table 1, some regional processes have been initiated. For example, the SADC Framework for Population Mobility and Communicable Diseases was developed in 2009 40 but remains in draft form with several member states refusing to ratify the Framework. The associated exercise to explore financing mechanisms – at the request of member states – was eventually completed by SADC in 2015, but remains unpublished (Oxford Policy Management, unpublished work). In 2010, the “SADC HIV and AIDS Cross Border Initiative” was awarded funding from the Global Fund to establish a regional cross‐border HIV programme involving the establishment of 32 clinics offering HIV testing and treatment, alongside primary care, in border areas and along transit routes to serve migrant and mobile populations, and local migration‐affected communities; 12 mainland member states signed Memorandums of Understanding (MoUs), agreeing to participate 41. However, progress is painfully slow: phase 1 involved just 12 clinics being opened, and the second phase – with a further 20 clinics due to open – was initiated at the end of 2017, with the anticipation that all 32 clinics will be handed over to member states in the first half of 2018. The slow process and challenges associated with this give a good indication of the challenges faced – both political and logistical – in developing and implementing cross‐border, migration‐aware HIV interventions at a regional level. The only regional health and migration policy process that has been implemented is the 2012 Declaration on Tuberculosis in the Mines 42; ratification of this Declaration happened quickly, and appears to be the result of any associated financial burden being the responsibility of the private sector, and not member states who are unwilling to commit to regional responses associated with migration and health.

### 2.3 A local lens: South Africa's engagement with migration and health

Within the SADC region, South Africa is the recipient of the largest number of cross‐border migrants (most of whom come from other SADC states), has a long history of internal migration, and a continued high prevalence and incidence of HIV 11, 13. However, responses to HIV that engage with population mobility are noticeably absent, in spite of calls being made for migration‐aware responses to HIV 10, 12, 43, 44. In addition, South Africa has not ratified the 2009 “Draft SADC Framework on Population Mobility and Communicable Diseases” – in spite of the development of financing models to support equitable cost‐sharing for regional responses to HIV. At a local level, some state‐led responses are in place, a notable example is the sub‐district of the Musina Municipality. Bordering Zimbabwe and home to the busiest border crossing in South Africa, Musina is a transit point for many. Local interventions include: mobile clinics providing antiretroviral therapy (ART) to migrant farmworkers; the Vhembe District Migrant Health Forum (MHF); and, Cross‐Border Forums on HIV and TB that involve partnerships with the Musina Municipality and their counterparts in Beitbridge – on the Zimbabwean side of the border 34, 69 (J Vearey and J Anderson, unpublished work).

Civil society and international organizations have attempted to step in to provide migration‐aware responses in spaces where the state is absent, including through HIV interventions supported by the International Organization for Migration (IOM) (J Vearey and J Anderson, unpublished work) 45, 46, 47, 69 and Medecins sans Frontiers 34. In addition, MHFs – networks of civil society, academic and government stakeholders – have been established in several areas of the country 48. Three national migration and health consultations have been held, in partnership between IOM, the National Department of Health and the University of the Witwatersrand 49, 50, 51. During the 2016 National Consultation, it was agreed that a National Migration and Health Forum needed to be established but, to date, no progress has been made 51. In 2014, a task team was established to explore HIV, migration and mobility within the National Strategic Plan for HIV, STIs and TB. However, responses to migration in the national HIV plan remain limited – and no framework has been developed for their implementation 12. South Africa is in the process of developing a National Health Insurance (NHI) which – in many ways – is a progressive development. However, current iterations of the NHI present a possible regression in the rights of non‐nationals to access healthcare, including ART 52.

## 3 Conclusions

There is an urgent need to develop migration‐aware 13 and mobility‐competent 3 responses to health globally. Particular concerns are raised around the need to develop appropriate responses to migration and HIV in SADC. While gains have been made in the response to HIV in SADC, multiple challenges remain – particularly in the era of UTT – as current responses do not adequately engage with migration and the movement of healthcare users 13, 53. HIV prevalence and incidence remain high, and it has been suggested elsewhere that it is the lack of migration‐aware programming that has undermined HIV prevention efforts at the regional level, where diverse internal and cross‐border population movements are prevalent 13. Currently, public healthcare systems in the southern African region are not designed to ensure continuity of care for migrant and mobile populations and prevailing xenophobic and anti‐foreigner sentiments present additional barriers to cross‐border migrants 11, 12, 34. The development of increasingly securitized migration management initiatives results in some cross‐border migrants struggling to access the documentation required to be in a country legally – which is often required to access healthcare 12. As a result, people moving both within countries and across national borders face barriers when trying to access HIV prevention, treatment and care 12, 53. The established evidence is clear: delayed testing and/or treatment initiation has negative impacts for infected individuals and for the populations with which they interact 34. By not engaging with migration, programmes currently designed to support HIV testing, antiretroviral treatment initiation and treatment continuity are jeopardized, with potentially, devastating consequences for HIV prevention programming – particularly in relation to UTT initiatives.

Concerns about how the Global Compact processes may undermine efforts to improve responses to health and migration globally should not be taken lightly. Vigilance is required to ensure that migration‐aware public health programming is not co‐opted to support securitization agendas that place the health and wellbeing of people on the move at risk. Globally, contextually appropriate migration‐aware responses to health are required; migration is a central public health imperative that provides a strategic opportunity to strengthen public health responses for all – including universal healthcare coverage 10, 12. In the SADC context, HIV prevention and treatment programmes will continue to struggle if migration is not mainstreamed, and key health targets will not be met 12. There is an urgent need to implement a regional strategy for the development of contextually appropriate migration‐aware responses to HIV in SADC, particularly in the UTT era. Efforts must be made to ensure that local‐level health programming – including HIV programming in SADC – is not undermined by current global moral panics, and resultant policy discourses.

## Competing interests

The author has no conflicts of interest to declare.
